# Onchocerciasis transmission status in some endemic communities of Cross River State, Nigeria after two decades of mass drug administration with ivermectin

**DOI:** 10.1038/s41598-023-31446-6

**Published:** 2023-04-03

**Authors:** Friday Maduka Chikezie, Kenneth Nnamdi Opara, Peace Mayen Edwin Ubulom, Clement Ameh Yaro, Rasha Khalifah Al-Akeel, Mike Yaw Osei-Atweneboana, Athanasios Alexiou, Marios Papadakis, Gaber El-Saber Batiha

**Affiliations:** 1 Department of Animal and Environmental Biology, University of Uyo, Uyo, Akwa Ibom State Nigeria; 2 Department of Zoology, Faculty of Entomology and Parasitology, King Saud University, Riyadh, Saudi Arabia; 3 Council for Scientific and Industrial Research-Water Research Institute, Accra, Ghana; 4 Department of Science and Engineering, Novel Global Community Educational Foundation, Hebersham, NSW 2770 Australia; 5 AFNP Med, 1030 Wien, Austria; 6 Department of Surgery II, University Hospital Witten-Herdecke, Heusnerstrasse 40, University of Witten-Herdecke, 42283 Wuppertal, Germany; 7 Department of Pharmacology and Therapeutics, Faculty of Veterinary Medicine, Damanhour University, Damanhour, AlBeheira, 22511 Egypt

**Keywords:** Ecology, Zoology, Diseases

## Abstract

Onchocerciasis is a parasitic disease transmitted by black flies. Human onchocerciasis is a public health and socioeconomic problem in Nigeria. Its prevalence and morbidity have reduced over the years because of control efforts especially, Mass Drug Administration with ivermectin. The current goal is to eliminate the disease transmission by 2030. Understanding the changes in transmission patterns in Cross River State is critical to elimination of onchocerciasis in Nigeria. This study was designed to determine the transmission dynamics of onchocerciasis in Cross River State after over two decades of mass ivermectin distribution in endemic communities. Agbokim, Aningeje, Ekong Anaku and Orimekpang are four endemic communities from three Local Government Areas of the State selected for this study. Transmission indices such as infectivity rates, biting rates and transmission potentials, parity rates and diurnal biting activities were determined. A total of 15,520 adult female flies were caught on human baits, Agbokim (2831), Aningeje (6209), Ekong Anaku (4364) and Orimekpang (2116). A total of 9488 and 5695 flies were collected during the rainy and dry seasons respectively in the four communities studied. The differences in relative abundance among the communities were statistically significant (*P* < 0.001). Monthly and seasonal fly numbers varied significantly (*P* < 0.008). There were differences in diurnal biting activities of flies in this study at different hours of the day and different months. The peak monthly biting rates were 5993 (Agbokim, October), 13,134 (Aningeje, October), 8680 (Ekong Anaku, October) and 6120 (Orimekpang, September) bites/person/month while the lowest monthly biting rates were 400 (Agbokim, November), 2862 (Aningeje, August), 1405 (Ekong Anaku, January) and 0.0 (Orimekpang, November and December) bites/person/month. Differences in biting rates among the study communities were significant (*P* < 0.001). The peak monthly transmission potential in Aningeje was 160 infective bites/person/month in the month of February while the lowest (except for months with no transmission) was 42 infective bites/person/month in the month of April. All other study sites had no ongoing transmission in this study. Transmission studies showed that there is progress toward transmission interruption especially in 3 out of the four studied areas. Molecular O-150 poolscreen studies is required to confirm the true transmission situation in the areas.

## Introduction

Onchocerciasis, also known as river blindness, is a neglected tropical disease (NTD) caused by *Onchocerca volvulus*, a filarial nematode parasite of man*.* Adult *Onchocerca* worms are found in subcutaneous nodules, where the female worms (during their 9–14 years of sexually active life) give birth to millions of microscopic young ones called microfilariae^1^. The microfilariae circulate in the peripheral blood under the skin from where they are picked up and transmitted to susceptible human hosts by susceptible female black flies of the genus *Simulium* during blood meal^2,3^. The immature worms then continue the process of development in susceptible black flies until they reach the infective L_3_ stage^4^.

In West Africa, there are nine documented vector species of the *Simulium damnosum* complex transmitting onchocerciasis. These species include *S. damnosum *sensu stricto*, S. sirbanum, S. dieguerense*, *S. konkourense*, *S. leonense, S. sanctipauli*, *S. soubrense, S. squamosum* and *S. yahense*^5–8^. The first three species are known as savannah flies which transmit the savannah strain of *O. volvulus,* causing the blinding, severe form of onchocerciasis while the rest belong to the forest group and transmit the forest strain of the parasite which causes more of skin disease^7,8^. In addition to their role as vectors of *Onchocerca volvulus,* black flies have also been implicated in the transmission of other parasites of man and animals^9–11^.

Regional onchocerciasis control in Africa started with the establishment of the Onchocerciasis Control Programme (OCP) in 11 participating countries in 1974 and later, the African Programme for Onchocerciasis Control (APOC) in 1995, covering 19 endemic countries in Africa^12^. The OCP initially relied heavily on vector control using larvicides. However, after the establishment of APOC and the arrival of ivermectin for onchocerciasis treatment, a mechanism for sustained delivery of doses of ivermectin (The community directed treatment with ivermectin, CDTI), was developed^13^, for mass drug administration using ivermectin. This strategy has been shown to reduce or break transmission of onchocerciasis in formerly endemic areas of the Americas, West, and East Africa^9,14–18^.

In West Africa, one of the most onchocerciasis endemic nations is Nigeria with approximately 3.2 million infections transmitted by black flies of the *Simulium damnosum* complex^11^. Documented onchocerciasis control efforts started in Nigeria in 1989 in some areas of the country, including parts of the present day Kwara, Kogi and Kaduna States^19^. However, nationwide treatment through the CDTI strategy started in the mid-1990s^20^.

Cross River State is one of the States in Nigeria that is endemic for onchocerciasis. Treatment with ivermectin started in the State in 1994 and by the year 2019, therapeutic coverage rose to 78% (FMOH, unpublished). Onchocerciasis epidemiological evaluations were conducted in 2009 and 2012 using skin snip analysis from children and adults in selected endemic communities. Prevalence of 2.84% and 7.51% were found in the 2009 and 2012 investigations respectively, showing that the State had ongoing transmission^21^. Another round of epidemiological evaluation was carried out in 2017 on dry blood spot (DBS) samples collected from children between the ages of 5–9 years of age from selected endemic communities using OV-16 ELISA analysis. The result this time showed a prevalence of 0.0% in this age group^20^. Despite these detailed epidemiological evaluations in the State, no corresponding entomological evaluation has been conducted in the State to determine the progress towards transmission interruption and elimination in line with WHO recommendations of acceptable annual biting rate (ABR) of 1000 bites/person/year and annual transmission potential (ATP) of 100/L3/person/year^4^; as well as those for interruption/elimination assessments^3,22^. This study addresses this knowledge gap after nearly 3 decades of control intervention. The study was designed to assess the degree of transmission of onchocerciasis in the selected areas after 27 years of community directed treatment with ivermectin (CDTI). This updated information is vital to achieving onchocerciasis interruption and elimination in the areas.

## Methods

### Study area

**Figure 1:**
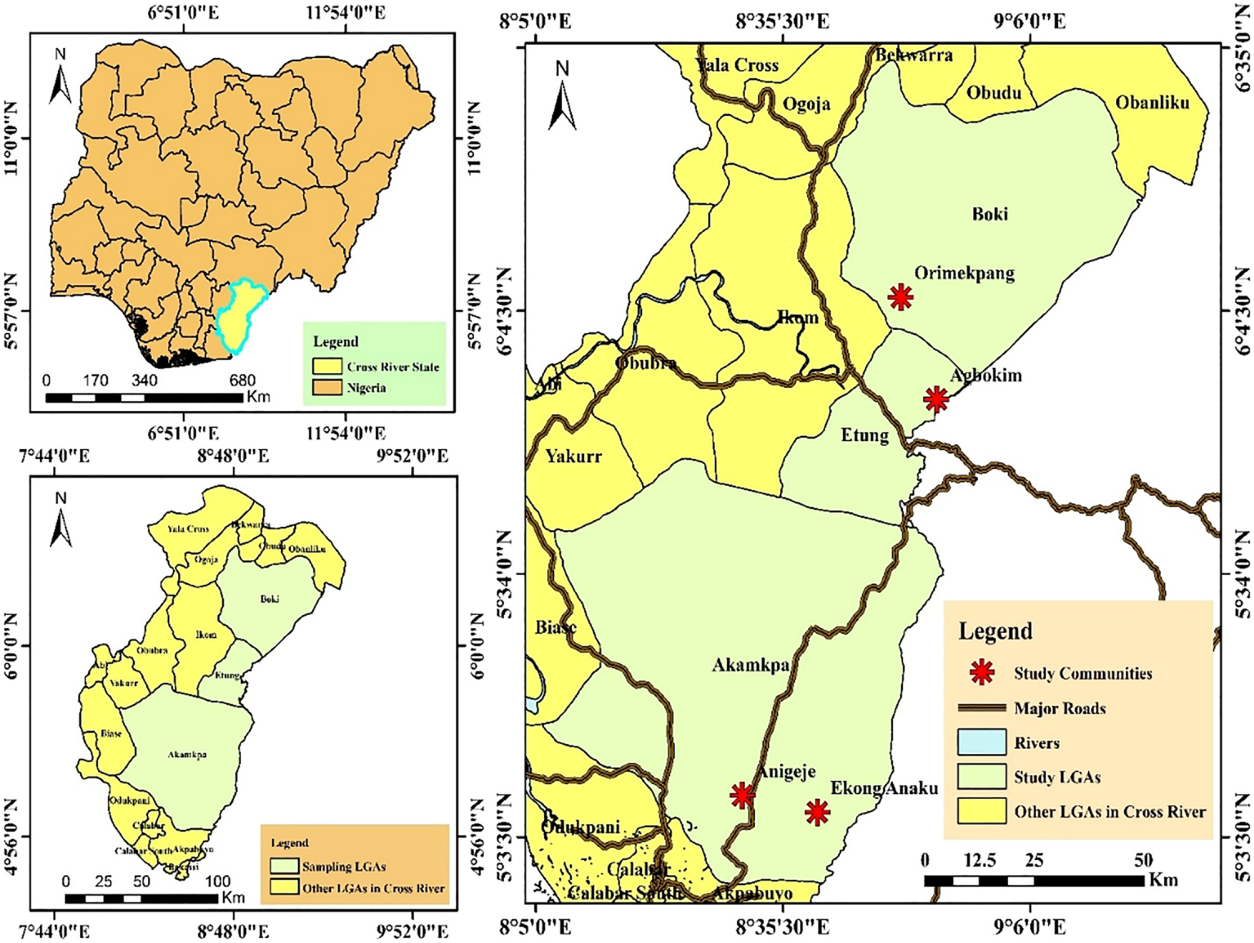
Map of the selected study sites in Cross River State detailing the main river systems and the location of breeding sites. Map was created by Clement Ameh Yaro using ArcMap 10.1 (ArcGIS Desktop: Release 10: Redlands, CA: Environmental Systems Research Institute).

The study was conducted in four randomly selected first-line endemic communities from three Local Government Areas (LGA) in Cross River State [Aningeje (Latitude 5.1389761, Longitude 8.50808) and Ekong Anaku (Latitude 5.10654, Longitude 8.66240833) in Akamkpa LGA, Agbokim (Latitude 5.90381, Longitude 8.90762) in Etung LGA and Orimekpang (Latitude 6.10132, Longitude 8.834505) in Boki LGA]. These were communities located immediately next to a breeding site with no other communities located between them and the breeding site^20^. Cross River State has two seasons, dry season (November to April) and rainy season (May to October). It shares boundaries with Benue State to the north, Ebonyi and Abia States to the west, and the Republic of Cameroon to the east. The southern border is with Akwa Ibom State as well as the Atlantic Ocean^23^. The Cross-River basin covers an area of about 70,000 km^2^, of which 50,000 km^2^ is in Cross River State, Nigeria, and the remaining 20,000 km^2^ is in Cameroon^24^. The Cameroonian section of the Cross-River basin is referred to as the Upper Cross River basin while the Nigerian section is the Lower Cross River basin based on some geographical parameters^25^. Figure 1 shows the main river systems and the location of *S. damnosum s.l*. breeding sites in the studied areas.

### Ethical approval and consent to participate

Ethical approval was obtained from the ethical review board of the Cross River State Ministry of Health with the reference number CRS/MH/HREC/020/VOL.V1/204. Advocacy visits were made to the communities prior to the commencement of the study. The relevance and benefit of the study were explained to the community leaders and all who would participate voluntarily. All fly collectors who had not been treated in the past six months preceding the study were treated by the community-based ivermectin distributor and properly documented before and after their involvement in the study.

All methods were carried out in accordance with relevant guidelines and regulations. Written informed consent were obtained from all the participants.

### Collection of human-biting adult black flies

The human landing catch method was used in collecting adult female black flies between June 2018 and May 2019, as described by^24,26,27^. Each site was sampled thrice a month between 7am and 6 p.m. by two fly collectors who worked alternately. The fly collectors sat on a wooden stool at the bank of the rivers and waterfalls, exposing their hands, and legs from their knees down. Any flies landing on the exposed parts of the body was collected before it could blood-feed by iverting a small collection tube over it and replacing the cap immediately with the fly in it^24^. Tubes containing collected flies were properly labelled to indicate site, time and date of collection. The total number of flies caught per day were recorded. Collected flies were transported from the sites in a cold box containing ice packs to prevent further development of any *Onchocerca* parasites present in the flies.

### Dissection and determination of parity rate

Live adult female flies were anaesthecized with chloroform and then dissected on a microscope slide containing a drop of physiological saline. Dissection began from the posterio-ventral end of the abdomen, from where the ovaries were pulled out for the determination of parity. The internal organs were closely observed and were recorded in order to determine whether the flies were nulliparous or parous. The nulliparous flies were those that had not taken any blood meal and had not completed any gonotropic cycle. As such, nulliparous flies could not have the parasite larvae. They have tightly coiled ovarian trachea, absence of follicular relics, absence of retained eggs as well as dark intestines. The parous flies were those that had taken at least one blood meal and had completed at least one gonotrophic cycle. These ones had follicular relics below maturing oocytes. Their ovary tracheal system are loosely stretched. They also have pale intestine, and may contain retained eggs. All parous flies were separated into head thorax and abdomen; and further dissected to search for the developmental stages of *Onchocerca* parasite in these anatomical parts. The number and various larval stages of *Onchocerca* species found in different parts of the flies were all noted and their stages of development at these sites recorded^28,29^.

### Entomological indices and their estimation

The monthly biting rates (MBRs) and the annual biting rates (ABRs) were measured as the theoretical number of *Simulium* bites received by a person who remains stationed at a catching point throughout the period of daylight for one complete month and one complete year respectively, in any given site. The monthly transmission potentials (MTPs) and the annual transmission potentials (ATPs) on the other hand were measured as the total number of infective bites (bites from adult flies bearing the third stage larvae of *Onchocerca* worm in their head) received in one month and in one year respectively, by an individual stationed at a catching point all through the period of daylight, in any given site^27^.

### Data analysis

Transmission indices (MBR, MTP, ABR and ATP) were estimated using results of manual dissection. The differences in these parameters were analyzed using the analysis of variance (ANOVA). The relative abundance of *S. damnosum* were also evaluated using ANOVA**.**

## Results

### Relative abundance of adult black flies

A total of 15,520 adult female flies were caught in the four communities throughout the study period. The highest number of flies, 6209 (40.01%) were caught in Aningeje while the least number of flies, 2116 (13.63%) were caught in Orimekpang. A total of 2831 (18.24%) and 4364 (28.12%) adult black flies were collected from Agbokim and Ekong Anaku respectively throughout the study period. Among the four sites, Aningeje recorded the highest number of flies during the rainy season as well as during the dry season. There were significant differences in fly numbers among the four study sites (*P* < 0.001). A total of 9488 and 5695 adult flies were caught during the rainy and dry seasons respectively. Comparison of monthly and seasonal variations in fly numbers across the study sites were statistically significant (*p* < 0.008). The monthly relative abundances of adult female black flies in the four study sites are presented in Tables 1, 2, 3 and 4.

**Table 1:** Population density and transmission indices of *S. damnosum* at Agbokim waterfall. [RSBR = Rainy season biting rate (May–October), DSBR = Dry season biting rate (November–April)].

Parameters	Months												Year total
	June	July	Aug	Sept	Oct	Nov	Dec	Jan	Feb	March	April	May	
Person’s day worked	3	3	3	3	3	3	3	3	3	3	3	3	36
Total flies caught	117	324	348	445	580	40	77	56	308	154	224	158	2831
Average daily catch/ person	39	108	116	148	193	13	26	19	103	51	75	53	79
No. of flies dissected	56	54	66	89	170	40	10	15	68	30	34	50	682
No. (%) of nulliparous flies	27 (48.2)	17 (31.5)	30 (45.5)	13 (14.6)	112 (65.9)	14 (34.8)	3 (30.0)	5 (33.3)	20 (29.4)	12 (40.0)	17 (50.0)	24 (48.0)	294 (43.1)
No. (%) of parous flies	29 (51.8)	37 (68.5)	36 (54.5)	76 (85.4)	58 (34.1)	26 (65.2)	7 (70.0)	10 (66.7)	48 (70.6)	18 (60.0)	17 (50.0)	26 (52.0)	388 (56.9)
Total no. (%) of flies infected	0 (0.0)	0 (0.0)	0 (0.0)	1 (1.1)	0 (0.0)	0 (0.0)	0 (0.0)	0 (0.0)	0 (0.0)	0 (0.0)	0 (0.0)	0 (0.0)	1 (0.3)
Flies (%) with L_1_ and L_2_	0 (0.0)	0 (0.0)	0 (0.0)	1 (1.1)	0 (0.0)	0 (0.0)	0 (0.0)	0 (0.0)	0 (0.0)	0 (0.0)	0 (0.0)	0 (0.0)	1 (0.3)
Flies (%) with L_3_	0 (0.0)	0 (0.0)	0 (0.0)	0 (0.0)	0 (0.0)	0 (0.0)	0 (0.0)	0 (0.0)	0 (0.0)	0 (0.0)	0 (0.0)	0 (0.0)	0 (0.0)
Total no. of L_3_ recorded	0	0	0	0	0	0	0	0	0	0	0	0	0
Monthly biting rates	1170	3240	3596	4450	5993	400	770	597	2877	1591	2240	1633	28,539
RSBR	20,182												
DSBR	8357												
MTP	0	0	0	0	0	0	0	0	0	0	0	0	0

### Diurnal biting activities of black flies

**Figure 2:**
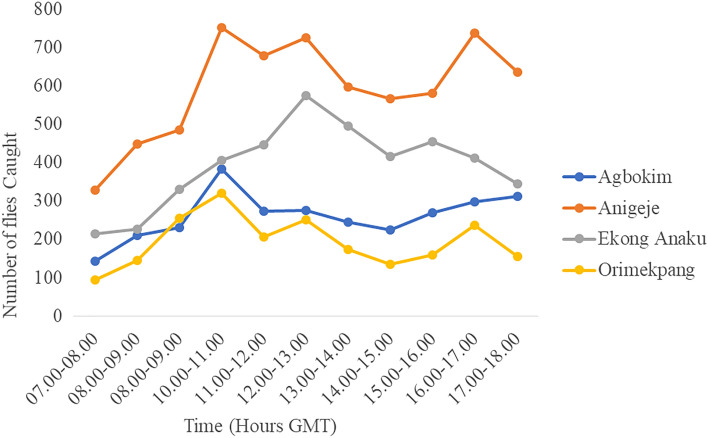
Diurnal biting rate of *S. damnosum* complex in the four study sites.

Hourly variations in fly biting activities were observed for the different sites. In Aningeje and Orimekpang, there was a trimodal pattern of biting activities of *Simulium* flies, with morning peaks occurring between 10 and 11 a.m., afternoon peak between 12 noon and 1 p.m. and evening peak between 4 and 5 p.m. At Agbokim, a unimodal pattern of black fly biting activity was observed between 10 and 11 a.m. with only one outstanding peak. Fly biting activities slowly declined towards the evening. At Ekong Anaku, a bimodal biting pattern was recorded with a pronounced peak between 10 and 11 a.m. and another less pronounced evening peak between 4 and 5 p.m. The hourly biting density of flies varied significantly across the sites **(***P* < 0.013). Figure 2 shows pooled hourly diurnal activity data of black flies for the various study sites throughout the study period.

### Parity rate

Parity rate in adult black flies collected from the various sites were similar though with minor variations. Agbokim consistently had above 50% parity during the rainy months, with exception of the month of September which had 44.7% parity. Parity during the dry months were also consistently over 50%. In Aningeje, it was only the month of June that recorded a parity rate of 40.3% among the rainy months. The rest of the study periods, including all the dry months recorded a parity rate of more than 50%. In Ekong Anaku, June and September had less than 50% parity among the rainy months, the rest were above 50%. Among the dry months, January alone had a parity rate of 42.5% while the rest were above 50%. In Orimekpang, only June had a 48.4% parity rate among the rainy months. The rest of the study periods, including all the dry months recorded a parity of more than 50% (Table 1, 2, 3 and 4). A comparison of the monthly parity rate among the communities and in the various months showed no significantly differences (*P* > 0.212).

### Transmission indices

#### Monthly biting rates

The monthly *Simulium* population density and onchocerciasis transmission indices at Agbokim (Agbokim waterfall) are shown in Table 1. A total of 2831 adult female flies were collected from this site. A total  682 (24.0%) were dissected, of which 294 (43.1%) were nulliparous while 388 (56.9%) were parous. Out of all the parous flies dissected, only 1 (0.03%) was infected with *Onchocerca* pre-infective larva. No infective fly nor fly with L_3_ larva in any part of the body was found from this site. The peak monthly biting rate of 5993 bites/person/month was recorded in Agbokim in the month of October while the lowest biting rate of 400 bites/person/month was recorded in the month of November. Monthly transmission potential was zero as no infective bites was recorded for this site. An annual biting rate of 28,539 bites/person/year was recorded in Agbokim. Annual transmission potential was also zero.

In Aningeje, the monthly *Simulium* population density and onchocerciasis transmission indices are shown in Table 2. A total of 6209 adult female flies were collected from the catching site at Aningeje, from which 1153 (18.6%) were dissected and 482 (41.8%) were nulliparous while 671 (58.2%) were parous. Out of all parous flies dissected, only 2 (0.03%) were infected with *Onchocerca* pre-infective larvae. Another 2 (0.03%) infective flies were recorded. The peak monthly biting rates of 13,134 bites/person/month was recorded in Aningeje (Kwa Falls) in the month of October. The lowest monthly biting rates of 2862, bites/person/month was found in the month of August. A peak monthly transmission potential of 160 infective bites/person/month was recorded in the month of February. Most of the sampling months recorded zero transmission potential, with no infective flies. The annual biting rate of black flies in Aningeje during the period of this study was 62,887 bites/person/year. On the other hand, an annual transmission potential of 297 infective bites/person/year was recorded in this study site.

**Table 2:** Population density and transmission indices of *S. damnosum* at Kwa falls (Aningeje). [RSBR = Rainy season biting rate (May–October), DSBR = Dry season biting rate (November–April)].

Parameters	Months												Year total
	June	July	Aug	Sept	Oct	Nov	Dec	Jan	Feb	March	April	May	
Person’s day worked	3	3	3	3	3	3	3	3	3	3	3	3	36
Total flies caught	434	385	277	595	1271	382	525	391	533	410	366	640	6209
Average daily catch/ person	145	128	92	198	424	127	175	130	178	137	122	213	172
No. of flies dissected	67	80	87	99	138	87	103	100	124	79	87	102	1153
No. (%) of nulliparous flies	40 (59.7)	29 (36.2)	42 (48.3)	49 (49.0)	64 (46.4)	24 (27.6)	38 (46.9)	46 (46.0)	41 (33.1)	31 (39.2)	33 (37.9)	45 (44.1)	482 (41.8)
No. (%) of parous flies	27 (40.3)	51 (63.8)	45 (51.7)	50 (51.0)	74 (53.6)	63 (72.4)	65 (63.1)	54 (54.0)	83 (66.9)	48 (60.8)	54 (62.1)	57 (55.9)	671 (58.2)
Total no. (%) of flies infected	0 (0.0)	0 (0.0)	0 (0.0)	1 (1.0)	1 (0.7)	0 (0.0)	0 (0.0)	0 (0.0)	1 (0.8)	0 (0.0)	1 (1.1)	1 (1.0)	5 (0.4)
Flies (%) with L_1_ and L_2_	0 (0.0)	0 (0.0)	0 (0.0)	1 (1.0)	0 (0.0)	0 (0.0)	0 (0.0)	0 (0.0)	0 (0.0)	0 (0.0)	0 (0.0)	1 (1.0)	2 (0.16)
Flies (%) with L_3_	0 (0.0)	0 (0.0)	0 (0.0)	0 (0.0)	1 (0.7)	0 (0.0)	0 (0.0)	0 (0.0)	1 (0.8)	0 (0.0)	1 (1.1)	0 (0.0)	3 (0.24)
Total no. of L_3_ recorded	0	0	0	0	1	0	0	0	4	0	1	0	6
Monthly biting rates	4340	3978	2862	5960	13,134	3820	5250	4040	4975	4237	3660	6631	62,887
RSBR	20,182												
DSBR	8357												
MTP	0	0	0	0	95	0	0	0	160	0	42	0	297

The monthly *Simulium* population density and onchocerciasis transmission indices at Ekong Anaku (Ekong Anaku River) are shown in Table 3*.* A total of 4364 adult female flies were collected from the Ekong Anaku River. Of this number, 917 (21.0%) were dissected and 390 (42.5%) were nulliparous while 527 (57.5%) were parous. None of all the parous flies dissected was infected with any of the various developmental stages of the *Onchocerca* worm*.* The peak monthly biting rate of 8680 bites/person/month was recorded in Ekong Anaku in the month of October. The lowest biting rate of 1405 bite/person/month was found in the month of January. The monthly transmission potential in this site was zero throughout the study period. The annual biting rate of black flies in the Ekong Anaku River during the period was 44,308 bites/person/year. Annual transmission potential in this study site was zero.

**Table 3:** Population density and transmission indices of *S. damnosum* at Ekong Anaku River. [RSBR = Rainy season biting rate (May–October), DSBR = Dry season biting rate (November–April)].

Parameters	Months												Year total
	June	July	Aug	Sept	Oct	Nov	Dec	Jan	Feb	March	April	May	
Person’s day worked	3	3	3	3	3	3	3	3	3	3	33	3	36
Total flies caught	182	302	636	290	840	372	489	136	222	207	369	319	4364
Average daily catch/ person	61	101	212	97	280	124	163	45	74	69	123	106	121
No. of flies dissected	23	118	140	68	122	50	80	40	56	76	75	69	917
No. (%) of nulliparous flies	14 (60.9)	40 (34.9)	68 (48.6)	35 (51.5)	51 (41.8)	18 (36.0)	26 (32.5)	23 (57.5)	22 (39.3)	31 (40.8)	29 (38.7)	33 (47.8)	390 (42.5)
No. (%) of parous flies	9 (39.1)	78 (66.1)	72 (51.4)	33 (48.5)	71 (58.2)	32 (64.0)	54 (67.5)	17 (42.5)	34 (60.7)	45 (59.2)	46 (61.3)	36 (52.2)	527 (57.5)
Total no. (%) of flies infected	0 (0.0)	0 (0.0)	0 (0.0)	0 (0.0)	0 (0.0)	0 (0.0)	0 (0.0)	0 (0.0)	0 (0.0)	0 (0.0)	0 (0.0)	0 (0.0)	0 (0.0)
Flies (%) with L_1_ and L_2_	0 (0.0)	0 (0.0)	0 (0.0)	0 (0.0)	0 (0.0)	0 (0.0)	0 (0.0)	0 (0.0)	0 (0.0)	0 (0.0)	0 (0.0)	0 (0.0)	0 (0.0)
Flies (%) with L_3_	0 (0.0)	0 (0.0)	0 (0.0)	0 (0.0)	0 (0.0)	0 (0.0)	0 (0.0)	0 (0.0)	0 (0.0)	0 (0.0)	0 (0.0)	0 (0.0)	0 (0.0)
Total no. of L_3_ recorded	0	0	0	0	0	0	0	0	0	0	0	0	0
Monthly biting rates	1820	3121	6572	2900	8680	3720	4890	1405	2075	2139	3690	3296	44,308
RSBR	26,389												
DSBR	17,919												
MTP	0	0	0	0	0	0	0	0	0	0	0	0	0

The monthly *Simulium* population density and onchocerciasis transmission indices at Orimekpang (Afi River) are shown in Table 4. A total of 2116 adult female flies were collected from the Afi River site. Out of this number, 366 (17.3%) were dissected and 146 (39.9%) were nulliparous while 220 (60.1%) were parous. No fly from this site was infected with any of the various developmental stages of the *Onchocerca* parasite*.* A peak monthly biting rate of 6120 bites/person/month was recorded in the month of September. Two consecutive months (November and December) had no flies. The monthly transmission potential in this site was zero throughout the period of this study. The annual biting rate of black flies in the Afi River during the period of this study was 22,215 bites/person/year while the Annual transmission potential was zero. There were significant differences in biting rates among the study areas (*P* > 0.001). The monthly biting rates in the various months were also significantly different (*P* < 0.004).

**Table 4:** Population density and transmission indices of *S. damnosum* at Afi river (Orimekpang). [RSBR = Rainy season biting rate (May–October), DSBR = Dry season biting rate (November–April)].

Parameters	Months												Year total
	June	July	Aug	Sept	Oct	Nov	Dec	Jan	Feb	March	April	May	
Person’s day worked	3	3	3	3	3	3	3	3	3	3	33	3	36
Total flies caught	99	121	225	612	465	0	0	101	174	78	81	100	2116
Average daily catch/ person	33	40	75	204	155	0	0	34	58	26	27	33	59
No. of flies dissected	31	43	35	43	77	0	0	0	52	18	27	40	366
No. (%) of nulliparous flies	16 (51.6)	16 (37.2)	17 (48.6)	20 (46.5)	29 (37.7)	0 (0.0)	0 (0.0)	0 (0.0)	14 (26.9)	7 (38.9)	11 (40.7)	16 (40.0)	146 (39.9)
No. (%) of parous flies	15 (48.4)	27 (62.8)	18 (51.4)	23 (53.5)	48 (62.3)	0 (0.0)	0 (0.0)	0 (0.0)	38 (73.1)	11 (61.1)	16 (59.3)	24 (60.0)	220 (60.1)
Total no. (%) of flies infected	0 (0.0)	0 (0.0)	0 (0.0)	0 (0.0)	0 (0.0)	0 (0.0)	0 (0.0)	0 (0.0)	0 (0.0)	0 (0.0)	0 (0.0)	0 (0.0)	0 (0.0)
Flies (%) with L_1_ and L_2_	0 (0.0)	0 (0.0)	0 (0.0)	0 (0.0)	0 (0.0)	0 (0.0)	0 (0.0)	0 (0.0)	0 (0.0)	0 (0.0)	0 (0.0)	0 (0.0)	0 (0.0)
Flies (%) with L_3_	0 (0.0)	0 (0.0)	0 (0.0)	0 (0.0)	0 (0.0)	0 (0.0)	0 (0.0)	0 (0.0)	0 (0.0)	0 (0.0)	0 (0.0)	0 (0.0)	0 (0.0)
Total no. of L_3_ recorded	0	0	0	0	0	0	0	0	0	0	0	0	0
Monthly biting rates	990	1250	2325	6120	4805	0	0	1042	1624	806	1600	1653	22,215
RSBR	17,143												
DSBR	5072												
MTP	0	0	0	0	0	0	0	0	0	0	0	0	0

#### Fly infectivity rates

The rainy season, recorded a total of 9825 adult flies, representing 63.3% of total catch. A total of 5695 flies were caught during the dry season, representing 36.7% of total number of flies collected. Out of all the flies dissected, 4 were infected in the rainy season and 2 in the dry season. The percentage infection rate was 0.04% and 0.03% for the wet and dry seasons respectively. Two out of the 4 infected flies recorded during the rainy season were infective while the other 2 were non-infective. For the dry season, the 2 infected flies were infective. A summary of transmission indices of *S. damnosum* in the four study communities is presented in Table 5.

**Table 5:** Summary of transmission indices of *S. damnosum* in the four study areas.

Characteristics	Agbokim	Anigeje	Ekong Anaku	Orimekpang
Person’ days worked	36	36	36	36
Total flies caught	2831	6209	4364	2116
Average daily catch per person	79	172	121	59
No. (%) of flies dissected	682 (24.1)	1153 (18.5)	917 (21)	366 (17.3)
No. (%) of parous flies	388 (56.9)	671 (58.2)	527 (57.5)	220 (60.1)
No. (%) of nulliparous flies	294 (43.1)	482 (41.8)	390 (42.5)	146 (39.9)
Total no. of flies infected (%)	1 (0.3)	5 (0.74)	0 (0)	0 (0)
Flies (%) with L_1_ and L_2_	1 (0.3)	2 (0.3)	0 (0)	0 (0)
Flies (%) with L_3_	0 (0)	3 (0.4)	0 (0)	0 (0)
Total number of L_3_ recorded	0	6	0	0

## Discussion

The result of this study has revealed that active transmission of onchocerciasis is still going on in Aningeje alone despite more than two decades of ivermectin intervention, and not in any of the three other studied areas of Cross River State. There is need to ascertain the treatment coverage in Aningeje and implement other elimination programmes to fast track the timely interruption of onchocerciasis transmission.

The presence of suitable aquatic habitat is an important factor influencing black fly population densities and distribution in the various studied areas. Breeding sites got flooded during the rainy season, flow rates and water levels increased. Turbidity also increased during the rains, negatively affecting the survival of immature black flies. Analogous situation was also reported by Chikezie et al.^30^ in Oji River area of Enugu State. However, the sheer increase in the number of breeding sites and rapids does explain the increase in fly populations during this season. Adult black fly populations increased during the rainy season as compared to dry season when water levels were low and most of the rapids, and by extension breeding sites had disappeared, in consonance with previous reports by^31–33^. Fly numbers in the studied areas varied both monthly and seasonally. More flies were caught during the rainy months and season than during the dry months and season. This finding agrees with the works of^24,32,34^. However, it contrasts with the findings of Mafiana^35^ and Adewale,^36^ who reported more flies during the dry season in some parts of southern Nigeria.

Marked variations in biting densities of flies were observed across the different study sites. This can be due to the geomorphological features of the breeding sites. For example, the two sites with the largest fly activities as adjudged by high fly numbers (Aningeje and Ekong Anaku) have very significant rocky substrata with submerged vegetations which usually break the flow of the waters and create rapids. This is the environment needed for the development and survival of the aquatic stages of *S. damnosum* s.l. These characteristics are true for the most productive sites. On the other hand, Agbokim River (emanating from the waterfall) in Agbokim and Afi River in Orimekpang lacked rocky substrata, at least at the areas that were feasibly prospected for immature stages. In fact, in Agbokim, the river is a large one, expanding to form a major transportation hub between Nigeria and the Republic of Cameroon. As such, there is high human commercial and agricultural activities that saw the river parameters required for black fly breeding greatly interrupted while on the other hand, creating a readily available source of blood meal for blood-seeking adult flies coming from nearby breeding sites (man-vector contact). The Afi River in Orimekpang is another large water body. Although there were not enough substrata observed to support breeding, vegetation by the river sides created minimal breeding sites and hence the low number of adult flies collected at these locations in comparison with the other sites studied. It is also possible that some of the flies collected during the study may have been migrant flies from nearby breeding sites.

The results of this study showed variations in daily biting activities of the simuliids in the various study areas. Agbokim had a unimodal fly biting activity with a peak at 10 a.m.–11a. m. The *Simulium* fly biting activity patterns reported in this study in Agbokim tallies with those of^37^, who reported a single peak of fly activity in a work in Liberia. It is also in tandem with the reports of^38,39^. In Ekong Anaku, a bimodal pattern of activity with peaks at 11 a.m.–12 noon and 3 p.m–4 p.m. was obtained. This finding agrees with several reports^24,35,36,40,41^. Aningeje had a trimodal peak of fly activities at 10 a.m.–11 a.m., 12 p.m.–1 p.m. and 4 p.m.–5 p.m. Orimekpang also had trimodal peaks of fly activities at 10am–11am, 12 p.m.–1 p.m. and 4 p.m.–5 p.m. Adeleke et al.^42^ also reported a similar trimodal fly biting pattern. Of all the sites, only Ekong Anaku conformed with the well-known bimodal pattern as have been reported by many authors^18,35,36,40,41^  elsewhere. However, the rest deviated from a bimodal pattern. The unimodal and trimodal patterns obtained in this study is in line with the report by Adeleke et al.^42^, who found unimodal and trimodal fly biting activities in the Osun River basin. Possible reasons for these findings might be due to differences in environmental factors obtainable in the various sites since these factors can differ from one site to another. These factors may include illumination pattern, temperature variations among others. Furthermore, ^2,36,43,44^ all confirmed that illumination, temperature, humidity, wind speed and other weather variables could result in the form of diurnal activities reported in the current study. This pattern of fly activity has an epidemiological implication as the peak biting periods corresponded to the peak of human outdoor activities such as farming, washing at the riverside and fishing, thereby increasing human-vector contact and potential parasite transmission.

Parity values in this study showed that more parous than nulliparous flies were caught. Parity were mostly above 50%. This agreed with previous studies by Chikezie et al.^45^ in Enugu where more parous flies were found than nulliparous flies. On the other hand, this report of more parous than nulliparous fly obtained in this study disagrees with the findings of^42^ who found more nulliparous flies in Osun River basin. This large parous proportions may suggest a high *Simulium* longevity or presence of migratory flies in line with the WHO^11^ report as well as that of^46^. The works of Renz^34^, Umeh et al*.*^47^, Opara et al.^41^ and Onah et al.^18^ reported that the presence of migratory flies could account for high parity rates. On the other hand, a low proportion of parous flies may be an indication of active breeding sites from where young flies are actively emerging. It may also be an indication of low fly longevity.

In this study, it can be deduced that both rainy and dry seasons recorded ongoing transmission. The dry season however recorded higher number of infective L_3_ stage indicating that even though fly numbers were less during this season, the few flies present were more likely to carry infective L_3_. The observation in the current study that relatively more infective L_3_ were found during the dry season than the rainy season as adjudged from results of fly dissection agrees with those reported by^36^ and^48^ who reported more infections during the dry season. This observation however is inconsistent with the results published by Renz^34^ and Opara et al.^24^ who found more infections during the rainy season. This differences may be accounted for by vector population sizes in the various seasons and locations of the study as well as treatment history. Population sizes in different locations may vary even in the same season. The monthly biting rates and transmission potentials in the current study were higher than the acceptable annual biting rate (ABR) and annual transmission potential (ATP) respectively, as stipulated by the WHO^4,49^. An ABR of 62,887 bites/person/year and ATP of 327.6 infective bites/person/year were recorded in Aningeje. Again, this value far exceeded the WHO threshold for ABRs and ATPs. The acceptable threshold values of ABR and ATP is 1000 bites/person/year for ABR and 100/L3/person/year for ATP^49^. With such high biting rates and transmission potentials, even low levels of infections in black fly may sustain transmission in a community.

There is a direct relationship between the presence and number of microfilariae in peripheral blood and subcutaneous tissues of the host and parasite transmission by the vectors. This is most likely an important factor that contributed to the low transmission status reported by the present study in Agbokim, Ekong Anaku and Orimekpang as vector black flies could not readily pick up the microfilariae (infective stage of *Onchocerca volvulus* to the black flies), which eventually will develop to L_3_ (the infective stage to man) within the blackfly vectors.

Annual and bi-annual treatment strategy for onchocerciasis is aimed at reducing the prevalence and intensity of onchocerciasis and to interrupt transmission of *O. volvulus*. From the result of the present study, Aningeje would likely benefit from bi-annual treatment to eliminate onchocerciasis. Sustaining a holistic bi-annual treatment with high treatment coverage could be the missing link towards transmission interruption and subsequent elimination in the areas. Even though molecular O-150 poolscreen analysis was not carried out, it is likely that the infections found in Aningeje are *O. volvulus*. This is likely, given findings in Cameroon which reported most infections in fly populations to be of *O. volvulus*^16^. Therefore, a holistic assessment of transmission using the recommended O-150 poolscreen is recommended to support or disavow the findings of the present study and inform the Nigerian Onchocerciasis Elimination Programme aright.

In line with the recommendation by Boatin et al.^50^, treatment with ivermectin when carried out in the beginning of the period of larger transmission will have a greater impact on suppressing transmission in Aningeje. Based on the results of this study, transmission in Aningeje was more in the dry season months of February and April. Bi-annual ivermectin treatment administered more preferably in June of the rainy season and January of the dry season could minimize parasite populations below certain threshold, even though infection rates may still be low around these months. Administering treatment in these months will effectively reduce microfilariae circulating in humans through to the peak of the transmission seasons. This suggestion is because optimal activities of ivermectin against *Onchocerca volvulus* occurs at about one month after administration^51–54^.

## Conclusion and recommendation

The results of the current studies showed that interruption of transmission of onchocerciasis in the study area is on track. The on-going transmission observed at Aningeje needs to further be investigated to ascertain the cause, especially the level of chemotherapeutic coverage. The need to commence twice a year mass ivermectin distribution in this area after confirmation of ongoing transmission by O-150 PCR poolscreen is strongly advocated in view of the 2030 road map for onchocerciasis elimination. Integration of mass drug administration (MDA) and vector control such as using slash and clear strategy would hasten the achievement of the elimination agenda. The infection rates and transmission potential of flies in this study was based on dissection. In view of the fact that these female flies, sometimes carry non-human *Onchocerca* parasites such as *O. ochengi*, which is morphological not easy to distinguish from *O. volvulus*, it is recommended that a detailed entomological evaluation involving PCR analysis of flies be conducted in Cross River.

## Data Availability

Data are made available upon reasonable request from the corresponding authors.
